# Impact of chronic administration of anabolic androgenic steroids and taurine on blood pressure in rats

**DOI:** 10.1590/1414-431X20165116

**Published:** 2016-05-31

**Authors:** A.E. Roşca, I. Stoian, C. Badiu, L. Gaman, B.O. Popescu, L. Iosif, R. Mirica, I.C. Tivig, C.S. Stancu, C. Căruntu, S.E. Voiculescu, L. Zăgrean

**Affiliations:** 1 Department of Physiology, Carol Davila University of Medicine and Pharmacy, Bucharest, Romania; 2 Victor Babeş National Institute of Research-Development in the Pathology Domain, Bucharest, Romania; 3 Department of Biochemistry, Carol Davila University of Medicine and Pharmacy, Bucharest, Romania; 4 R&D Irist Labmed, Bucharest, Romania; 5 Department of Endocrinology, C.I. Parhon National Institute of Endocrinology, Carol Davila University of Medicine and Pharmacy, Bucharest, Romania; 6 Department of Neurology, Colentina Clinical Hospital, Carol Davila University of Medicine and Pharmacy, Bucharest, Romania; 7 Department of Lipoproteins and Atherosclerosis, N. Simionescu Institute of Cellular Biology and Pathology, Bucharest, Romania

**Keywords:** Anabolic androgenic steroids, Nandrolone decanoate, Taurine, Blood pressure, Angiotensin-converting enzyme, Nitric oxide

## Abstract

Supraphysiological administration of anabolic androgenic steroids has been linked to increased blood pressure. The widely distributed amino acid taurine seems to be an effective depressor agent in drug-induced hypertension. The purpose of this study was to assess the impact of chronic high dose administration of nandrolone decanoate (DECA) and taurine on blood pressure in rats and to verify the potentially involved mechanisms. The study was conducted in 4 groups of 8 adult male Wistar rats, aged 14 weeks, treated for 12 weeks with: DECA (A group); vehicle (C group); taurine (T group), or with both drugs (AT group). Systolic blood pressure (SBP) was measured at the beginning of the study (SBP_1_), 2 (SBP_2_) and 3 months (SBP_3_) later. Plasma angiotensin-converting enzyme (ACE) activity and plasma end products of nitric oxide metabolism (NO_x_) were also determined. SBP_3_ and SBP_2_ were significantly increased compared to SBP_1_ only in the A group (P<0.002 for both). SBP_2_, SBP_3_ and ACE activity showed a statistically significant increase in the A *vs* C (P<0.005), and*vs* AT groups (P<0.05), while NO_x_ was significantly decreased in the A and AT groups *vs* controls (P=0.01). ACE activity was strongly correlated with SBP_3_ in the A group (r=0.71, P=0.04). These findings suggest that oral supplementation of taurine may prevent the increase in SBP induced by DECA, an effect potentially mediated by angiotensin-converting enzyme.

## Introduction

Anabolic androgenic steroids (AAS) are synthetic cholesterol derivatives of testosterone, designed for clinical applications in conditions such as hypogonadism, breast cancer, anemia, and various chronic catabolic states (1). Due to their anabolic effects, they are currently being used in high doses among athletes to enhance performance or in the bodybuilding community for cosmetic reasons. AAS abuse has been blamed for a large number of adverse side effects, including liver and kidney toxicity, endocrine dysfunction, psychiatric and behavioral disturbances and a variety of significant alterations on cardiovascular system with potentially severe complications (hypertension, atherosclerosis, left ventricular dysfunction, thromboembolic events, life-threatening arrhythmia, and sudden cardiac death) (2,3). The continuous increase of androgen misuse and its relationship to premature death has regarded AAS abuse as a major medical and public health issue (1,4). Although there is a considerable amount of evidence depicting the whole spectrum of detrimental effects of androgen abuse, data describing their association with elevated blood pressure or with the development of hypertension are still ambiguous and inconclusive (3,4). Therefore, our study was designed to generate more data concerning the influence of supraphysiological administration of AAS on blood pressure in rats, and to underline possible mechanisms that may be involved.

Taurine, often referred to as a conditionally-essential amino acid, and present in high concentrations in the brain, retina, liver, bile, myocardium, kidney, skeletal muscles and blood cells, has many beneficial effects, such as anti-oxidative, anti-inflammatory and anti-apoptotic activity, membrane ion exchange modulation, osmoregulation, bile acid conjugation, and neurotransmission regulation (5-7). There are multiple animal and human studies describing the protective role of taurine on the heart and vessels, suggesting that it could be useful in the prevention of cardiovascular disease (6). This sulfur-amino acid has showed several key actions on cardiovascular risk factors. It improves lipid profile, decreases plasma homocysteine, and reduces the inflammation and oxidative injuries of the intima layer of vessels, leading to a delay in the initiation and progression phases of atherosclerosis (5). Taurine also provides cardioprotection, exerts modulator effects on hemostasis, has a protective action in diabetes and its complications, and shows a beneficial influence on blood pressure (6,8). Additionally, we have previously reported protective effects of taurine against the hypercoagulable state induced by suprapharmacological nandrolone decanoate (DECA) administration in rats (9,10). Later, Ahmed et al. reported that taurine, by virtue of its anti-inflammatory, antioxidant and anti-apoptotic effects, prevented testicular toxicity and DNA damage induced by high doses of DECA administration in rats (11). Although taurine is also recognized for a large number of favorable actions on vasculature and it appears to be effective as a depressor agent in hypertensive situations, the involved mechanisms are not entirely clear and are still being debated (6,12).

In this study, we assessed the impact of taurine administration on blood pressure, independently or in combination with high doses of DECA, and we pointed out the possible mechanisms that may underline their effects in rats. We hypothesized that this sulfur-amino acid could alleviate the blood pressure response induced by the supraphysiological DECA administration. The present experiment is the first to assess the influence of a simultaneous treatment with these two drugs on blood pressure.

## Material and Methods

### Animals

The study was conducted in 32 adult male Wistar rats, aged 14 weeks, with body weight 323±27 g (mean±SD), housed in individual cages, floored with wood shavings, in a room with constant temperature (23°C) and 12-h light-dark cycle (lights on at 7:00 am), with access to rat chow and water *ad libitum*. For economic and ethical reasons, we used the minimum number of animals needed to achieve the scientific objectives. All animal procedures were carried out in accordance with the guiding principles for biomedical research involving animals, as stated by the European Communities Council Directive #86/609/EEC and with approval of the Ethics Committee for Animal Research of the Carol Davila University of Medicine and Pharmacy (Bucharest, Romania).

### Chemicals

DECA was purchased from Norma Hellas Pharmaceutical Industry (Greece, 2 mL Vial, 100 mg/mL). Taurine (crystalline powder) was obtained from Merck KGaA (Germany).

### Experimental design

The animals were randomly assigned to four treatment groups (n=8 for each group) - androgen group (A): rats received a weekly high dose of DECA (10 mg/kg body weight), diluted in sesame oil with benzyl alcohol (90:10, v/v) as vehicle (15 mg/mL), by intragluteal injection, for 12 weeks; taurine group (T): rats were supplemented with 2% (159.8 mM) taurine in drinking water for the same time period; animals in this group also received a weekly intramuscular injection of sesame oil with benzyl alcohol (90:10, v/v); androgen and taurine group (AT): rats treated with both DECA and taurine, in similar doses to those used in the aforementioned groups; control group (C): rats were given only weekly intramuscular vehicle injection. The DECA dose was chosen according to previous studies and was comparable to the dose that has been reported as being frequently used by abuser athletes, 600 mg/week, or approximately 8 mg·kg^-1^·week^-1^ (13).

Noninvasive measurements of systolic blood pressure (SBP) were performed at the beginning of the study (SBP_1_), 2 (SBP_2_), and 3 months later - at the end of the study (SBP_3_).

Since rat blood sampling throughout the study could interfere with blood pressure assessment and influence the results, we decided to perform the biochemical measurements only at the end of the study. The rats were euthanized by withdrawing blood from the heart, under induced and maintained anesthesia, after 12 weeks of treatment. Blood samples withdrawn by cardiac puncture were collected into heparinized plastic tubes.

### Noninvasive blood pressure measurement

Systolic blood pressure was measured by tail-cuff plethysmography technique, following the BIOPAC User Guide instructions (Non-invasive Small Animal Tail Blood Pressure Systems - NIBP200A, BIOPAC Systems, Inc., USA). To condition the animal before starting the BIOPAC recordings, rats were subjected to a training session. They were placed in a suitable body restrainer several times a day, for 3 days, accompanied each time by the tail warming procedure. For accurate pressure measurement, prior to obtaining each recording, the animals were placed in the restrainer for at least 10 to 15 min and the tail was warmed to 32°C. Also, a proper room temperature was maintained and a proper animal handling was carefully carried out. A darkened nose cone was placed over the proximal extremity of the rodent restrainer to limit the animal's view and reduce stress level. However, if the animal was under stress and moved the tail steadily, it was taken out of the restrainer and allowed to relax and become inactive before resuming the measurements and obtaining a successful SBP reading. SBP was recorded between 12:00 am and 2.30 pm. As a generally accepted method, 9 SBP measurements were performed for each rat and mean values were calculated. The lowest stable consecutive values were taken to avoid the influence of animal restlessness on SBP reading.

It would probably have been useful to assess blood pressure in physically trained rats, for an experimental model of AAS abusing athletes. However, this would have excluded other situations associated with high plasma levels of circulating androgens, such as endocrine diseases. Additionally, the model would not have reflected AAS abuse for cosmetic purposes in untrained subjects.

### Plasma angiotensin-converting enzyme (ACE) activity

A direct spectrophotometric assay for determining the plasma ACE activity in rat serum was used. The substrate used is a furan-acryloyl-blocked tripeptide, 2-furan-acryloyl-phenylalanyl-glycyl-glycine (FAPGG), which undergoes an 80 nm blue shift in the absorption spectrum upon hydrolysis into FA-Phe and Gly-Gly. ACE activity assays were made at 340 nm and 37°C using a measuring time of 10 min. One unit (U) of ACE activity was expressed as the amount of the enzyme that will hydrolyze 1 µmol of the substrate FAPGG into FAP and glycyl-glycine in 1 min at 37°C (14). Normalized plasma ACE activity was determined at the end of the measurements, by dividing the ACE value to total plasma protein value for each animal. Total protein was assayed by Bradford method (Bio Rad Laboratories, USA). Plasma samples were diluted from an estimated initial total protein concentration of 7 g/dL (70 mg/mL). Absorbance was measured spectrophotometrically at 595 nm against a standard containing bovine serum-albumin.

### Plasma stable end products of NO metabolism (nitrate and nitrite)

Nitrate and nitrite (NO_x_) analysis was performed spectophotometrically by an assay that combines reduction of nitrate with vanadium (III) and measurement of nitrite in a single step. Reactions were carried out at 37°C, taking into consideration that at temperatures below 80°C, nitrate reduction by vanadium (III) is suspended following nitrite formation. Griess reagents [N-1-naphthylethylenediamine dihydrochloride (NEDD) and sulphanilamide (SULF)] were used as trapping agents for simultaneous detection of nitrate and nitrite. A nitrate standard solution (50 µL) was serially diluted (from 100 to 5 µM) in duplicate in a 96-well flat-bottomed, polystyrene microtiter plate (Corning, USA). After loading the plate with samples (50 µL), addition of a saturated solution of VCl_3_ (40 mg in 5 mL 1 M HCl) to each well (50 µL) was immediately followed by addition of SULF 2% in 1 M HCl (25 µL) and 0.1% NEDD in dH_2_O (25 µL). The samples were incubated for 40 min at 37° C, and nitrate content was measured spectrophotometrically at 540 nm. The VCl_3_ solution and the Griess reagents were freshly prepared immediately prior to application to the plate. Sample blank values were obtained by substituting diluting medium for Griess reagent. Nitrite was similarly measured, except that samples and nitrite standards were only exposed to Griess reagents (15).

### Statistical analysis

The normal probability distribution was tested using the Kolmogorov-Smirnov test. Continuous data are reported as means±SD. One-way ANOVA followed by Tukey's *post hoc* analysis was performed for comparisons between groups regarding biochemical parameters and body weight. General linear model (repeated measures) was used to compare the intergroup and intragroup variations of blood pressure, followed by Bonferroni's *post hoc* test. The relationship between different parameters was assessed by Pearson's method. SPSS (Statistical Package for Social Sciences, Inc., USA) Windows 20.0 software was used for statistical analysis. A two-sided P<0.05 was considered to be statistically significant.

## Results

Body weight was similar in the four groups of rats at the beginning of the experiment (P=0.55). At the end of the study the increase in body weight was smaller in the A, T and AT groups (Table 1), although the differences were not significant from the controls (P=0.06).

**Table:**
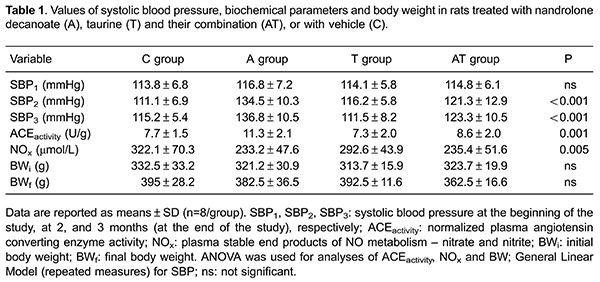

### Effects of DECA and taurine on blood pressure

**Figure 1:**
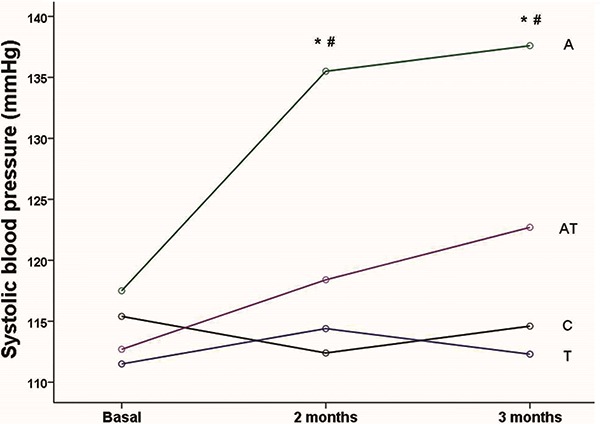
Effect of nandrolone decanoate (A), taurine (T) and their combination (AT) on systolic blood pressure (SBP) of rats. There was a significant increase of SBP at 2 and 3 months *vs* basal SBP only in the A group. SBP was significantly higher in the A *vs* control (C) group and also in the A *vs* AT group (n=8 for each group), at 2 and 3 months (General Linear Model; repeated measures; and Bonferroni's *post hoc* test). *P≤0.002 *vs* basal blood pressure;^#^P≤0.007 *vs* the C and AT groups.

During the experiment, systolic blood pressure significantly changed only in the A group (intragroup variation). In this group, SBP_2_ and SBP_3_ significantly increased compared to SBP_1_ (SBP_2_ *vs* SBP_1_, P=0.002, SBP mean difference = 5.9 mmHg, 95% confidence interval (CI) = 1.95–9.84 and SBP_3_ *vs* SBP_1_, P<0.001, SBP mean difference=7.52 mmHg; 95%CI =3.48–11.56) (Figure 1).

A significant difference was found in SBP among the 4 groups of rats (intergroup variation) at 2 and 3 months after the beginning of the study (P<0.001). *Post hoc* analysis showed that SBP significantly increased in the A *vs* C group, at both 2 (P=0.001, SBP mean difference = 12.6 mmHg; 95%CI=3.99–21.21) and 3 months (P<0.001, SBP mean difference = 16.07 mmHg; 95%CI=8.75–23.39). A significant difference was also found in the A *vs* AT group at both 2 (P=0.007, SBP mean difference = 10.95 mmHg; 95%CI=2.34–19.56) and 3 months (P<0.001, SBP mean difference = 12.27 mmHg; 95%CI=4.95–19.59). No significant differences were found in SBP between the T and C groups, or between the AT and C groups, at 2 or 3 months (P>0.93; Figure 1).

### Effects of DECA and taurine on plasma ACE activity

**Figure 2:**
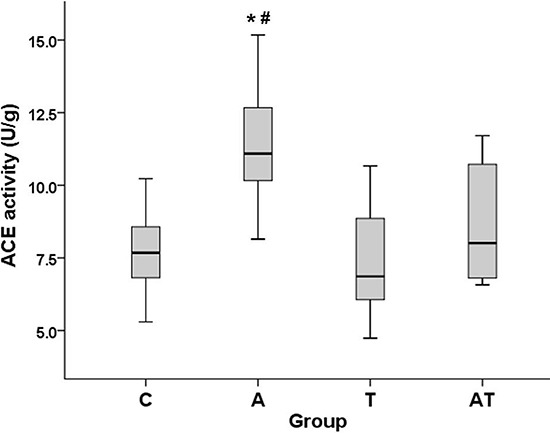
Effect of nandrolone decanoate (A), taurine (T) and their combination (AT) on plasma angiotensin converting enzyme (ACE) activity in rats. There was a significant increase of ACE activity in the A *vs* control (C) group and also *vs* the AT group (n=8 for each group). The box-and-whisker plots report the minimum, 25% percentile, median, 75% percentile, and maximum values. *P=0.004 *vs* C group; ^#^P=0.04 *vs* AT group. (one-way ANOVA and Tukey's *post hoc* analysis).

A significant difference was found concerning ACE activity (P=0.001, Table 1). *Post hoc* analysis revealed a statistically significant increase in the A *vs* C group (P=0.004) and in the A *vs* AT group (P=0.04), while between the AT and C groups, or C and T groups there were no significant differences (Figure 2). In the A group ACE activity was strongly correlated with SBP_3_ (r=0.71, P=0.04), but not with SBP_2_. ACE activity also tended to be related to SBP_3_ in the AT group, but without reaching statistical significance (r=0.63, P=0.08).

### Effects of DECA and taurine on plasma stable end products of NO metabolism (nitrate and nitrite)

**Figure 3:**
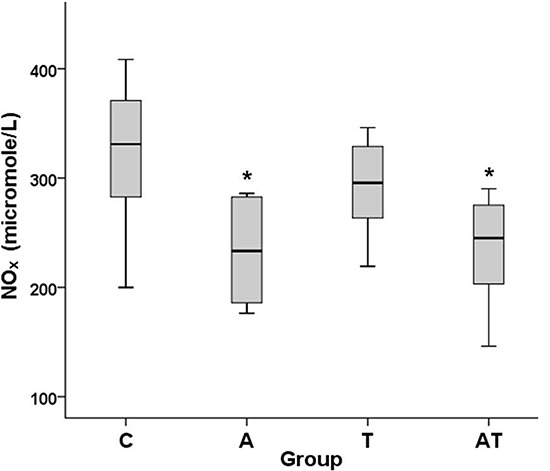
Effect of nandrolone decanoate (A), taurine (T) and their combination (AT) on plasma levels of stable end products of NO metabolism - nitrate and nitrite (NO_x_) - in rats. There was a significant decrease of NO_x_ in the A and AT groups *vs* control (C) group (n=8 for each group). The box-and-whisker plots report the minimum, 25% percentile, median, 75% percentile, and maximum values. *P=0.01 *vs* the C group (one-way ANOVA and Tukey's *post hoc* analysis).

Mean values for plasma levels of NO stable metabolic end products - nitrate and nitrite - are reported in Table 1. There was a significant difference concerning NO_x_ (P=0.005). *Post hoc* analysis of NO_x_ showed a significant decrease in the A and AT groups *vs* the C group (P=0.01), while no significant differences were registered between the A and AT groups, or between the C and T groups (Figure 3). There were no significant correlations between NO_x_ and SBP_2_ or SBP_3_ in any group.

## Discussion

The present study brings complementary data regarding the action of DECA and taurine on blood pressure, a field of interest still dominated by controversial data. It also shows the cumulative effect of the two drugs on blood pressure for the first time. We found that chronic, suprapharmacological DECA administration induced a pressor response in rats, which was prevented by concomitant taurine administration. DECA also increased ACE activity and decreased NO_x_ levels, suggesting that it may partially contribute to blood pressure elevation in the androgen treated group. Chronic taurine supplementation in drinking water had no effect in intact rats, but significantly decreased ACE activity in the mixed treated group, thus offering a potential explanation for the blood pressure change in this group of rats.

### Effects of DECA on blood pressure

Our findings related to the influence of supraphysiological AAS administration on noninvasively measured systolic blood pressure support those of several other animal studies (16,17). In humans, increased blood pressure, or even hypertension has been linked to high levels of circulating androgens, induced either by various endocrinological diseases [polycystic ovary syndrome (PCOS), adrenogenital syndrome, virilizing gonadal tumors, enzyme defects, medication induced hyperandrogenism] (18,19), or by AAS abuse in athletes or amateur bodybuilders (1,2). However, there is still a controversy regarding the pressor effects of AAS misuse in the sporting environment (3,4). The currently available data, coming mostly from observational studies, indicate either an elevation of blood pressure (which can persist for up to 1 year after drug intake cessation) (2,20,21) or no significant blood pressure changes in AAS users (3,22,23). The variety of outcomes may result from the lack of homogeneity in type, dosage, and timing of AAS administration, exercise regimen and from the difficulty in controlling drug abuse in both the AAS and control groups (1).

The increase of plasma ACE activity in the DECA treated group is also consistent with published data. It has been previously proven that androgens have a stimulatory action on renin-angiotensin system (RAS), upregulating the angiotensinogen and renin mRNA expression, increasing angiotensin II and plasma renin activity, and enhancing the angiotensin type 1 receptor pathways (24-26). Moreover, high levels of plasma androgens increased the renal cortical expression of ACE in a rat model of PCOS (26). DECA, at doses twice the one we used, 20 mg·kg^-1^·week^-1^, was shown to induce an elevation of cardiac tissue ACE activity in untrained rats (16). The close correlation between ACE activity and SBP_3_ identified in our study allowed us to consider the ACE pathway as one of the possible mechanisms underlying SBP variation in the DECA group.

Regarding the plasma stable end products of NO metabolism, nitrate and nitrite, the decrease of NO_x_ level in the DECA group could also explain the increase of the SBP in this group*vs* controls. These findings are consistent with previous data. It was proven that in humans supraphysiological doses of androgens could decrease the endothelial NO synthase (eNOS) expression (27). Also, DECA administered in a dose commonly used by heavy AAS abusers was shown to decrease the level of aortic eNOS in rats (28). On the other hand, there are data supporting the view that androgens may have a stimulatory effect on NO production, as was noted in experiments performed on the rat Leydig cell culture or testicular tissue sample (11,29). A possible explanation for plasma NO decrease exerted by androgens, as obtained in the present study, has been given by Reckelhoff et al. (24) and afterwards by Xue et al. (25), who promoted the opinion that androgens could induce blood pressure elevation by superoxide concentration increase. The cause of this increase is the enhancement of the oxidative stress generated by androgens, either directly or indirectly, via RAS stimulation and angiotensin II release. Increased superoxide anion can subsequently interact with NO, leading to peroxynitrite formation, one of the most potent oxidative compounds and a substance with a powerful vasoconstrictor action induced through multiple mechanisms (24,25). Considering all the existent controversial data and hypothesis, our study brings an additional argument to the detrimental action of AAS on NO production when administered in supraphysiological doses. This could reflect the presence of endothelial dysfunction, one of the cardiovascular side-effects often reported following AAS misuse.

### Effects of taurine on blood pressure

As it is revealed by the noninvasive pulse plethysmographic assessment, taurine exerted no influence on systolic blood pressure in intact rats, but significantly decreased its level at 2 and 3 months after administration in the mixed treated animals. This effect is consistent with the antihypertensive action of sulfur-amino acid, evidenced in animal studies using hypertensive models, and suggested by several clinical and epidemiological studies (5,6,8). On the other hand, accumulating evidence from laboratory experiments showed that oral taurine supplementation does not seem to affect blood pressure in healthy animals (6). The described inhibitory effect of the sulfur-amino acid in the hypertension setting and the neutral effect in intact animals may be of worth, as taurine was able to stabilize the disorder and further preserve the homeostasis. In fact, based on its protective role on the cardiovascular system, taurine has been approved in Japan as a therapeutic agent for heart failure treatment (30).

Taurine was effective in lowering the increased level of ACE activity induced by nandrolone, while exerting no action in intact rats. A tendency for correlation has been found between ACE activity and SBP_3_ in the mixed treated group. Our findings didn't establish a causative mechanism by which taurine exerts its benefits, but one possible explanation for the blood pressure reduction in the mixed treated group of rats might be its action on ACE. Several lines of evidence support our results related to taurine influence on ACE activity. It has been previously proven that taurine attenuates renin-angiotensin system overactivity, preventing blood pressure elevation induced by renin, potentiating the effects of ACE inhibitors, modulating the expression of AT_2_ receptors, and antagonizing the harmful effects of angiotensin II on the heart, blood vessels and kidneys (8,30,31). However, scarce data point out the influence of the sulfur-amino acid on altered ACE activity caused by several pathological conditions (32,33). In these studies taurine exerted no influence on ACE activity in intact rats, as we also found. This can be assigned to the generally observed behavior of the sulfur-amino acid, to act only on drug-induced organ or systemic pathophysiology (34).

Taurine presented a slight propensity to decrease plasma NO stable end products in intact rats, without reaching statistical significance. The neutral influence of taurine on NO metabolism in healthy animals has been cited in the literature (11,32), despite the fact that a vasodilator effect of the sulfur-amino acid has been systematically proven (6). The also neutral effect on plasma NO_x_ levels obtained in the mixed treated group is quite surprising, considering the reduction of blood pressure in this group. This is even more striking when taking into account the considerable amount of available data concerning the stimulatory action of taurine on NO production (5,6). On the other hand, data from experiments conducted on animal models or cell cultures also reveal an inhibiting action of sulfur-amino acid on NO production (35-37). It has been suggested that this influence may be linked to the taurine's ability to quench NO, which along the proved inhibitory effect on superoxide generation, can prevent reactive nitrogen species formation - i.e., peroxynitrite (11,37). The antioxidant activity of the sulfur-amino acid is actually quite well stated (5,31). Recently, taurine has been shown to exhibit protective effects against heavy exercise-induced nitrosative inflammation and stress, preventing inducible nitric oxide synthase (iNOS) expression in rats skeletal muscle (38), which may be of interest considering the stimulatory action of supraphysiological doses of AAS on oxidative stress. Overall, the influence of taurine on the process of NO synthesis and release is still controversial. The current contradictory data are probably dependent on the large variety of study designs. In this context, complementary data regarding this field of investigation could be valuable.

The present findings led us to the conclusion that taurine may have the ability to protect against a panel of insults generated by AAS administration, but it fails to exhibit any influence on healthy animals. Oral supplementation of the sulfur-amino acid restored the nandrolone-induced increased values of SBP. This effect may be explained by the detrimental influence of DECA on ACE and NO_x_, and by the beneficial influence of taurine on ACE. Our study brings complementary data into a very exciting area of interest, which still has a knowledge gap and controversy over the possible mechanisms through which the two drugs may influence blood pressure. Extrapolating to humans, this study challenges the assumption that taurine, a substance safely used for more than two decades, may be useful in several circumstances associated with high levels of circulating androgens. It remains to be established if this sulfur-amino acid may have an effective role in individuals abusing of AAS, with poor compliance to standard antihypertensive therapies and to the physician's AAS withdrawal recommendation. This could be of interest especially because taurine is often used to enrich beverages with the purpose of boosting athletic performance. Certainly, prospective studies with long-term taurine use are needed in order to verify these hypotheses and to clarify the exact mechanisms that may underline these effects.
